# Development of an Antibiotic Resistance Breaker to Resensitize Drug-Resistant *Staphylococcus aureus*: *In Silico* and *In Vitro* Approach

**DOI:** 10.3389/fcimb.2021.700198

**Published:** 2021-08-16

**Authors:** Gopalakrishnan Thamilselvan, Hema Bhagavathi Sarveswari, Sahana Vasudevan, Alex Stanley, Karthi Shanmugam, Pothiappan Vairaprakash, Adline Princy Solomon

**Affiliations:** ^1^Quorum Sensing Laboratory, Centre of Research in Infectious Diseases, School of Chemical and Biotechnology, SASTRA Deemed to be University, Thanjavur, India; ^2^Department of Bioinformatics, School of Chemical & Biotechnology, SASTRA Deemed to be University, Thanjavur, India; ^3^Department of Chemistry, School of Chemical & Biotechnology, SASTRA Deemed to be University, Thanjavur, India

**Keywords:** NorA, efflux Pump, *Staphylococcus aureus*, bioisostere, boron acid, antimicrobials

## Abstract

Efflux pumps are one of the predominant microbial resistant mechanisms leading to the development of multidrug resistance. In *Staphylococcus aureus*, overexpression of NorA protein enables the efflux of antibiotics belonging to the class of fluoroquinolones and, thus, makes *S. aureus* resistant. Hence, NorA efflux pumps are being extensively exploited as the potential drug target to evade bacterial resistance and resensitize bacteria to the existing antibiotics. Although several molecules are reported to inhibit NorA efflux pump effectively, boronic acid derivatives were shown to have promising NorA efflux pump inhibition. In this regard, the current study exploits 6-(3-phenylpropoxy)pyridine-3-boronic acid to further improve the activity and reduce cytotoxicity using the bioisostere approach, a classical medicinal chemistry concept. Using the SWISS-Bioisostere online tool, from the parent compound, 42 compounds were obtained upon the replacement of the boronic acid. The 42 compounds were docked with modeled NorA protein, and key molecular interactions of the prominent compounds were assessed. The top hit compounds were further analyzed for their drug-like properties using ADMET studies. The identified potent lead, 5-nitro-2-(3-phenylpropoxy)pyridine (5-NPPP), was synthesized, and *in vitro* efficacy studies have been proven to show enhanced efflux inhibition, thus acting as a potent antibiotic breaker to resensitize *S. aureus* without elucidating any cytotoxic effect to the host Hep-G2 cell lines.

## Introduction

Antibiotics are the first class of drugs that have been effective in treating many infections of clinical importance (Fernandes, 2006). However, the widespread usage and cross-reactivity of these antibiotics have enabled bacteria to gain resistance against most of the drugs through their evolutionary mechanisms (Tomoari et al., 2014). Consequently, treatment of bacterial infections is becoming a challenge due to the emergence of multidrug resistant organisms (MDROs) (Tugal et al., 2015). Among the MDROs is methicillin-resistant *Staphylococcus aureus* (MRSA), a major cause of hospital-acquired (nosocomial) and community-acquired infections (Hartmann et al., 2010). Also, the complexity increases in the treatment of MRSA with standard therapies as it exhibits resistance to the drugs of the latest resort such as tetracyclines, aminoglycosides, and fluoroquinolones (Marquez, 2005; Sabatini et al., 2012). MRSA exhibits a diversity of resistance mechanisms against antimicrobials that either reduce or prevent their transport into the cell or efflux them from the cell with multidrug resistant transporters (MDR pumps) (Sangwan et al., 2008). Among known resistance mechanisms, MDR pumps are transport proteins involved in the extrusion of toxins from the bacterial cells and show resistance to a wide range of antimicrobial compounds as well as host-derived microbicidal compounds thus enabling the bacteria to thrive in their ecological niche (Sun et al., 2014; Blair et al., 2015). To date, 14 MDR pumps have been described for *S. aureus*, including NorA, NorB, MedA, QacA/B, and Smr (Costa et al., 2013). The NorA efflux pump that belongs to the major facilitator superfamily (MFS) is a significant contributor to antibiotic resistance as it can expel a wide range of chemical probes, such as ethidium bromide (EtBr), acriflavine, quaternary amine compounds, fluoroquinolones, rhodamine-6-G, puromycin, and chloramphenicol (CHL), and confers resistance (Markham et al., 1999). Also, NorA shares sequence homology with other MDR pumps, which led to a hypothesis that it may possess a large hydrophobic binding pocket with a wide range of substrate specificity and so an attractive novel drug target for the identification of efflux pump inhibitors (EPIs) to fight against resistance (Neyfakh, 2002; Poole and Lomovskaya, 2006; Cloete R. et al., 2018). Several non-antibiotics such as reserpine, verapamil, flavones, isoflavones, porphyrin pheophorbide A, and acylated glycosides are reported to be NorA inhibitors that act as selective small-molecule modulators rather than clinical drug candidates (Schillaci et al., 2017). In this context, the efforts are focused on the design and synthesis of novel chemotypes, which could synergize with ciprofloxacin (CIP) against *norA* overexpressing *S. aureus* strains and impact on clinical relevance (Sabatini et al., 2011; Sabatini et al., 2013; Astolfi et al., 2017; Felicetti et al., 2017). Based on the recent study, chemicals with boronic acid entities are proven to be the potential inhibitors of the NorA efflux pump and reduce the antibiotic concentration fourfold (Fontaine et al., 2015). On a closer look to the synthesis protocol, the synthesis of chemicals having boron species requires the use of dangerous lithium reagents (Leermann et al., 2011), magnesium reagents (Clary et al., 2011), and more expensive palladium and iridium catalysts (Jiang et al., 2016). Several literary works also support the use of lithium, palladium, and iridium catalysts for the synthesis procedure (Sharp and Snieckus, 1985; Sharp et al., 1987; Zhdankin et al., 1999; Das et al., 2003; Billingsley and Buchwald, 2008; Hafner et al., 2016). To overcome the challenges of toxicity and high cost involved, the current work undertakes a classical medicinal chemistry approach of bioisostere replacement. Bioisosterism allows molecularly modifying the lead compound without compromising the drug target interaction, enhances the biological activity with improved pharmacokinetics, and reduces toxicity (Patani and LaVoie, 1996). In this regard, the current work aims to design new molecules from the reported lead compound by replacing the boronic acid moiety with a small molecule chemotype capable of restoring CIP activity on *S. aureus* strains by inhibition of NorA using SwissBioisostere (Wirth et al., 2013). This study combines systematically the interdisciplinary aspect of bioinformatics, medicinal chemistry, and microbiological studies to design and validate a promising NorA EPI.

## Materials and Methods

### NorA Efflux Pump: 3D-Structure Prediction and Quality Assessment

The efflux pump, NorA, is a hydrophobic protein having 12 putative transmembrane domains. As the name suggests, the NorA efflux pump is used for the transport of fluoroquinolones, resulting in the limited efficacy of the antibiotic. As the experimentally determined 3D structure of the NorA efflux pump is not yet publicly available, we attempted to predict its structure using a theoretical approach. The primary amino acid sequence of NorA of *S. aureus* (accession number: Q5HHX4) with 388 amino acids (42.324 Da) was downloaded from UniProtKB (www.uniprot.org). The 3D structure of the downloaded NorA sequence was predicted using the I-TASSER (Iterative Threading ASSEmbly Refinement) server (https://zhanglab.dcmb.med.umich.edu/I-TASSER/). I-Tasser is a hierarchical protocol for automated protein structure prediction. A detailed systematic approach is used to generate the atomic structure models through multiple thread alignments, iterative assembly simulations, and finally, atomic-level structure refinement. The I-TASSER output is the C-score, a confidence score for estimating the quality of predicted models. The C-score is typically in the range of [-5, 2], where a C-score of a higher value signifies a model with high confidence and *vice versa*. We obtained the C-score of model-1 = -0.13, model-2 = 0.43, model-3 = -0.26, model-4 = -3.01, and model 5 = -3.40 for the top 5 models obtained from I-TASSER. Based on the C-score, we have chosen model-2 for further quality assessments. The stereochemical quality of model-2 was further evaluated using the Ramachandran plot. The plot made between the phi and psi angles of model-2 showed that 98.2% of the residues are in the favored region, and 1.8% of the residues are in the disallowed region. Since the C-score and the stereochemistry of model-2 were good and acceptable, we have chosen them for further molecular docking studies.

### Bioisosteric Replacement

Earlier studies reported that boronic species were found to inhibit the NorA efflux pump and potentiate ciprofloxacin activity (Fontaine et al., 2014). In the present study, we derived biologically equivalent replacements (bioisosteres) for the chosen parent chemotype, 6-(3-phenylpropoxy)pyridine-3-boronic acid, by replacing the boronic acid (-B(OH)_2_) functional group with other equivalent bioisosteres using the SwissBioisostere (http://www.swissbioisostere.ch) web-server interface (Wirth et al., 2013). The SwissBioisostere database contains information on 21,29,355 data points, corresponding to ~5,586,482 unique replacements, which are mapped to their biological performance in biochemical assays. The biological performance corresponds to 1,948 molecular targets and 30 target classes. This allows bioisosteric modifications of boronic acid in 6-(3-phenylpropoxy)pyridine-3-boronic acid. The 2D structure of 6-(3-phenylpropoxyl)pyridine-3-boronic acid was drawn using MarvinSketch 6.2 embedded in the SwissBioisostere server. Using the “Fragment 1” window in the SwissBioisoster, the boronic acid functional group (-B(OH)_2_) was kept intact, and the remaining part of the structure was grouped and labeled as the -R group and queried against the database. This search enables SwissBioisoster to identify biologically equivalent functional groups through the detection of matched molecular pair and mining bioactivity data in the ChEMBL database. It gives an output of an extensive overview of which replacements for the queried substructure (-B(OH)_2_) have been observed in the database. There are 42 equivalent replacements for the boronic acid that were identified by the webserver. It also provides the observed frequency, activity difference distribution, success-based score, or chemical similarity between the fragments.

### Ligand Preparation

The 2D structures of all the 42 bioisosteric replacements obtained through SwissBioisosters were drawn using MarvinSketch (v17) (https://chemaxon.com/products/marvin). Efficient and accurate 2D to 3D conversion is a key precursor to any computational analyses. We used the LigPrep module implemented in Schrodinger for preparing the ligands. LigPrep generates accurate energy minimized 3D molecular structures and eliminates mistakes in ligands to reduce downstream computational errors. All the 42 compounds were saved as a single sdf file and prepared using the LigPrep wizard. OPLS-2005 forcefield was used for energy minimizing and optimizing the structures. We did not expand tautomeric and ionization states and stereoisomers for the input structures, as it may influence the bioisosteric replacements.

### Protein Preparation

Any successful structure-based modeling research demands an accurate starting structure. Hence, the 3D structure of NorA protein modeled using I-TASSER (model-2) was prepared using protein preparation wizard implemented in Schrodinger (Sastry et al., 2013). Our protein preparation briefly includes adding missing hydrogens, determining optimal protonation states for histidine residues, optimizing protein’s hydrogen bonding network, and restraining energy minimization. The prepared protein was further used for molecular docking studies.

### Binding Site Prediction and Receptor Grid Generation

The accuracy of any molecular docking studies depends on how precisely the binding sites are defined (Friesner et al., 2006). In the absence of an experimentally solved co-crystallized structure of NorA with known inhibitors, we used Schrodinger SiteMap for predicting the binding sites of the modeled NorA protein (model-2). The highest-ranked binding site (Sitecore = 1.119) was selected as the putative NorA binding site, and Ile23, Pro24, Leu26, Pro277, Phe 47, Arg98, Val144, Tyr225, and Gly348 were set as binding site residues. The selected binding site residues agree with some of those residues reported in previous studies (Kalia et al., 2012). The binding site for molecular docking was defined using the receptor grid generation wizard implemented in Schrodinger. The grid box was kept cantered on the binding site amino acids identified using Sitemap. The size of the box was set to default. We visually inspected and confirmed that all the key binding site amino acids are within the defined grid box.

### Molecular Docking

Molecular docking study was carried out using the Glide XP (extra precision) algorithm implemented in Schrodinger. Glide XP reliably finds the correct binding modes and outperforms other docking programs in receiving lower RMS deviations from the native-crystallized structure. All the 42 ligands were docked onto the binding site of the modeled NorA protein. The docking results were analyzed based on the glide score and the binding interactions. The protein–ligand complex interactions were visualized using PyMOL (https://pymol.org/2/).

### ADMET Predictions

Accurate prediction of ADMET properties before expensive experiments on preclinical and clinical studies is essential to reduce the failure of the drug in the later stages. We used Schrodinger QikProp for evaluating the drug-likeness of the identified lead molecules. QikProp predicts the widest variety of pharmaceutically relevant properties including AlogP, PSA, hepatotoxicity, CYPD26 inhibition, aqueous solubility, blood–brain barrier penetration, and human oral absorption, Initially, the newly generated lead molecules through bioisosteric replacements were filtered for Lipinski’s rule of 5 (molecular weight ≤ 500 Da, lipophilicity ≤ 5, hydrogen bond donor ≤ 5, hydrogen bond acceptor ≤ 10). Those molecules that satisfied Lipinski’s rule of 5 were further screened for Veber’s rule (rotatable bonds < 10 Å and polar surface area < 140 Å). The most important ADMET descriptors were calculated for the molecules that satisfied Veber’s rule (Lipinski et al., 2001; Shinzato et al., 2020).

### Synthesis of the Selected Bioisostere, 5-Nitro-2-(3-Phenylpropoxy)Pyridine

5-Nitro-2-(3-phenylpropoxy)pyridine (5-NPPP) was synthesized by treating (3-bromopropyl)benzene with 5-nitropyridin-2-ol in a basic medium provided by K_2_CO_3_ in acetonitrile solvent. The reaction was carried out at 80 °C for 2 h.

 
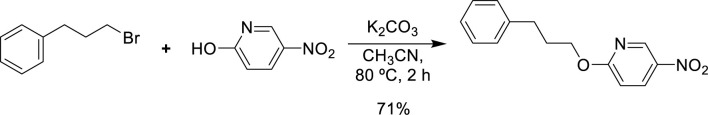


In a typical synthesis, a mixture containing 5-nitorpyridin-2-ol (1.00 g, 7.14 mmol), (3-bromopropyl) benzene (1.71 g, 8.57 mmol, 1.31 mL), potassium carbonate (1.4 g, 10.1 mmol), and acetonitrile (5.0 mL) was stirred at 80 °C for 2 h. Then, the reaction mixture was filtered, and the filtrate was concentrated. The resulting mass was purified by column chromatography to obtain 5-NPPP as a yellow-colored solid (1.3 g, 71% yield). The compound was characterized by NMR spectroscopy. ^1^H NMR (300 MHz, CDCl_3_) *δ* 2.17 (qt, *J* = 7.5 Hz, 2H), 2.74 (t, *J* = 7.5 Hz, 2H), 4.02 (t, *J* = 7.5 Hz, 2H), 6.54 (d, *J* = 9.9 Hz, 1H), 7.18-7.34 (m, 5H), 8.06 (dd, *J_1_* = 9.9 Hz, *J_2_* = 3.0 Hz, 1H), 8.48 (d, *J* = 3.0 Hz, 1H); ^13^C NMR (75 MHz, CDCl_3_) *δ* 29.9, 32.6, 50.8, 119.6, 126.4, 128.2, 128.7, 130.5, 133.0, 139.3, 139.8, 161.4; ESI-MS obsd 259.1079, calcd 259.1077 [(M + H)^+^,M = C_14_H_14_N_2_O_3_].

### Bacterial Strains and Growth Conditions

*S. aureus* clinical isolates (*Sa*-P1987, *Sa*-P1920, *Sa*-AB459, *Sa*-P2003, *Sa*-Eye33, *Sa*-P2017, *Sa*-AB77, and *Sa*-P1995) were received from J.S.S Medical College, Mysore, and ATCC 29213, SA-1199B, and SA-1199 were used for the study. The *S. aureus* strains were retrieved from glycerol stocks and were maintained in Trypticase Soy Agar (TSA; HiMedia; Mumbai; India) at 37° C.

### Chemicals and Antibiotics

Antibiotics (vancomycin (VAN), cefoxitin (CEF), norfloxacin (NOR), cephalexin (CEP), ciprofloxacin (CIP), CHL, azithromycin (AZI), cloxacillin (CLO), EtBr, and Verapamil) were purchased from Sigma-Aldrich Chemical Co. (St. Louis, MO). The stock solutions of antibiotics and inhibitors were prepared with sterile water. In the case of reserpine, dimethyl sulfoxide (DMSO) was used for the preparation of the initial stock solution and further diluted to desired concentrations with water or culture broth (Aeschlimann et al., 1999).

### Resistance/Susceptible Profiling of Selected *S. aureus* Isolates

The antibiotic resistance/susceptibility assay was carried for both the standard and clinical isolates by the broth microdilution method according to CLSI guidelines (Wikler, 2006). The selected antibiotics, VAN (16 μg ml^-1^), CEF (8 μg mL^-1^), NOR (16 μg ml^-1^), CEP (32 μg ml^-1^), CIP (128 μg ml^-1^), CHL (30 μg ml^-1^), AZI (32 μg ml^-1^), and CLO (128 μg ml^-1^), were used for MIC determinations as interpreted in the susceptibility interpretive criteria reported in the appropriate CLSI tables.

### Correlation of MDR With Overexpression of Efflux Pump, NorA

*S. aureus* clinical isolates grown overnight in TSB 37 °C were diluted to 100-fold in fresh TSB. The broth was incubated at 37 °C with shaking at 250 rpm for 3 h (OD_600_ of ∼0.6). RNA protect bacteria reagent (Qiagen) was added to the cell culture and left at room temperature for 30 min. Cell pellets were collected and lysed as described in the RNAprotect Bacteria Reagent Handbook (Lan et al., 2010). Total RNA was extracted by following the manufacturer’s guidelines of the HiMedia RNA Extraction Kit (MB613). The integrity and purity of the extracted RNA were verified using agarose gel electrophoresis and NanoDrop (Thermo Scientific, Waltham, USA), respectively. Total RNA was converted to cDNA using the iScript™ cDNA Synthesis Kit using the manufacturer-recommended protocol. Relative quantification of NorA gene expression was performed with *gmk* (guanylate kinase) as the housekeeping gene, using the fast-real-time PCR system (Eppendorf, India) (**Table S1**). The resistance pattern of the selected strains was correlated with the cycle threshold (CT) of *norA* as it is inversely proportional to its expression level. The strain that showed the lowest CT value was scored for its overexpression of NorA and chosen for further study, and it was confirmed with the EtBr efflux assay (Schmittgen and Livak, 2008; Balasubramanian et al., 2020; Tinoush et al., 2020).

### Determination of Minimum Inhibitory Concentration of 5-NPPP

The minimum inhibitory concentration (MIC) of the bioisostere, 5-NPPP, was determined against the *norA* overexpressed clinical isolate, *Sa*-P2003. The strain was grown at 37° C in Mueller–Hinton Broth and diluted until it reached a cell density of 2.9*10^8^ CFU ml^-1^. Varying concentrations of the 5-NPPP (1 to 1,000 μg mL^-1^) were tested against the growth of the clinical isolate, *Sa*-P2003, and SA-1199B. After 24 h, the growth of the cells was recorded at 595 nm using a 96-well microtiter plate reader (BioRad i-Mark). The MIC of 5-NPPP is defined as the lowest concentration that inhibits the growth by ≥ 90% (for MIC_90_) or ≥ 50% (for MIC_50_) when compared to control (untreated) (Rodríguez-Tudela et al., 2003).

### Determination of Minimum Effective Concentration of 5-NPPP

The overnight diluted bacterial suspension of *Sa*-P2003 and SA-1199B was plated in 96-well microtiter plates containing varying concentrations of 5-NPPP (0.5 to 0.125 µg ml^-1^) and CIP (0.25–8 µg ml^-1^). The culture condition was maintained at 24 h at 37 °C, and after 24 h, the growth inhibition was evaluated by measuring the OD_595_. The untreated bacterial culture was taken as a negative control. The percentage inhibition of the combination and individual treatments of cultures were calculated to score minimum effective concentration (MEC). Furthermore, the 5-NPPP and CIP interaction was analyzed by fitting the data in a zero interaction potency (ZIP) model using the R-package synergy finder (https://bioconductor.org/packages/release/bioc/html/synergyfinder.html).

### EtBr Efflux Assay

For the EtBr efflux assay, NorA overexpressing *Sa*-P2003 and *S. aureus* SA-1199B cultures as independent trials were suspended to an OD of 0.2 in buffer (50 mM NH_4_Cl, 110 mM NaCl, 52 mM Tris base, 7 mM KCl, 0.4 mM Na_2_HPO_4_, and 0.2% glucose) with pH adjusted to 7.5. The cells were loaded with a dose of 10 μg ml^-1^ EtBr as it does not exert any cytotoxic effect. Then, the EtBr-loaded cells were centrifuged at 12,000 rpm for 5 min, and then, pelletized cells were resuspended in EtBr-free PBS buffer with 0.4% glucose supplemented either with or without 5-NPPP (0.25 μg ml^-1^). A known NorA inhibitor, verapamil (25 μg ml^-1^), was used as a positive control. The real-time EtBr efflux from the cells was observed at every 5-min intervals for 30 min in triplicates. The intensity of fluorescence was measured at 530 nm for excitation and 600 nm for emission, respectively, using a Jasco FP-8200 spectrofluorometer (Jasco Corporations, Japan) (Brenwald et al., 1998).

### Cytotoxicity Assays

Hep-G2 (human liver carcinoma) was purchased from NCCS, Pune and cultured in an RPMI medium at 37°C under 5% CO_2_ supplemented with 10% fetal bovine serum (FBS), 100 U ml^-1^ penicillin, and 100 μg ml^-1^ streptomycin. The cell viability was assessed by the reduction of the dye, 3-(4,5-dimethylthiazol-2-yl)-2, 5-diphenyltetrazolium bromide to purple formazan crystals by mitochondrial succinate dehydrogenase enzyme in living cells. The cells were seeded into 96-well plates at a concentration of 10^4^ cells/well and allowed to incubate for 24 h. Once the cells reached a subconfluent stage, then they were incubated with the bioisostere, 5-NPPP, at its 1XMIC, 10X, and 100X MIC for 48 h. After incubation, 10 μL of MTT (5 mg ml^-1^ in PBS) was added to the microtiter plate and was incubated for 4 h at 37° C. The MTT was removed, and 100 μL DMSO was added to each well to solubilize formazan crystals. Plates were incubated at 37° C for 20 min, and the absorbance was read at OD_590_. Experiments were performed in triplicates, and the percentage cell viability was calculated (Kakhki et al., 2014).

### Statistical Analysis

The results were expressed as mean ± SD. Statistical analysis was performed using the GraphPad Prism software version 8 (GraphPad Software Inc., San Diego, CA, United States). Significance was checked with the Dunnett *t*-test for multiple comparisons and paired Student’s *t*-test (p ≤ 0.05) (Balasubramanian et al., 2020).

## Results

### Structural Prediction and High Throughput Virtual Screening of NorA Inhibitors Using the Bio-Isosteric Approach

**Figure 1:**
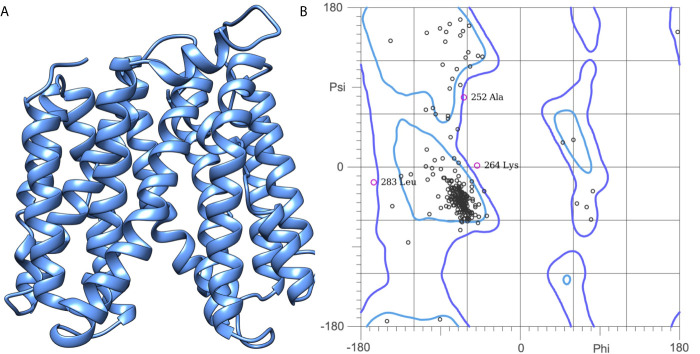
**(A)** Construction of the NorA efflux pump model: A three-dimensional model of NorA was generated using I-TASSER. The best scoring model (C-score = 0.43; TM Score = 0.70 ± 0.12) was visualized using UCSF Chimera. **(B)** Quality prediction of modeled NorA protein using the Ramachandran plot. The structure of NorA protein is found to span in the regions of beta-sheets and right-handed alpha helix. Phi and Psi represent the torsion angles.

**Figure 2:**
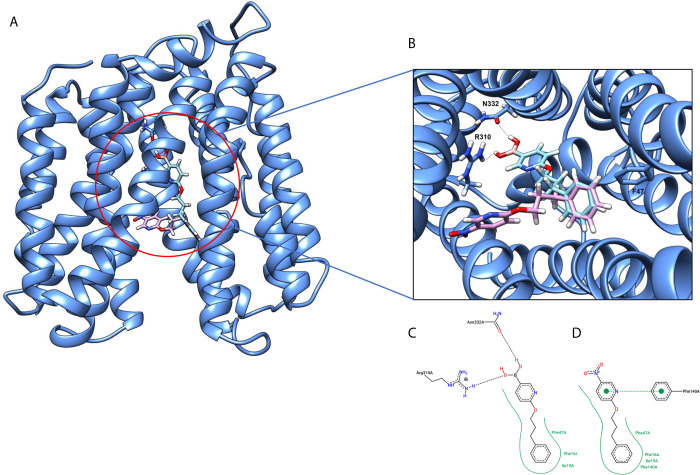
**(A)** Hypothetical binding mode of 5-NPPP at the hydrophobic cleft of the NorA efflux pump (blue). The ligand is represented in a multicolor stick model. **(B)** The binding interactions of 6-(3-phenylpropoxy) pyridine-3-boronic acid and 5-NPP with the binding site amino acids. **(C)** 2D-interaction map of 6-(3-phenylpropoxy) pyridine-3-boronic acid with the NorA efflux pump. **(D)** 2D interaction map of 5-NPPP with the Nor A efflux pump in hydrophobic cleft with Phe47, Phe16, Phe140, and Ile19 enables a stronger hydrophobic interaction due to the closer proximity of the ligand with these residues as well as possessing π–π interactions.

The 3D structure of *S. aureus (strain COL)* Quinolone resistance protein NorA sequence (PDB Accession no: Q5HHX4) (**Figure 1**) was modeled and validated by a Ramachandran plot. The molecular interactions of the generated 42 compounds with the modeled NorA protein were analyzed using molecular docking. The docked complexes were further subjected for binding free energy calculations using Molecular mechanics/Generalised Born Surface Area (MMGBSA) (**Table 1**), and the higher the negative value the stronger the interaction and the higher the binding affinity. Out of these 42 compounds, 19 compounds displayed binding energies that ranged between −6.07 and −7.2 kcal/mol indicating a greater potential for the ligand to bind to NorA. This greater binding potential exertion was followed by another 20 compounds that showed binding energies from −4.0 to −5.9 kcal/mol. MMGBSA calculations of the receptor–ligand complex showed that the complexes formed between the NorA and ligands are highly stable (**Table 1**). Investigation of the interaction between 5-NPPP was analyzed based on the docking scores. The ligand 5-NPPP was positioned within the transmembrane alpha helixes of the modeled NorA. The parent chemotype binds within hydrophobic cleft having residues Ile19A, Phe47A, and Phe16A, while the 5-NPPP binds with Ile19A, Phe47A, Phe16A, and Phe140A (**Figure 2**).

**Table 1:** Identification of lead compound through screening of small molecules based on docking and activity scores.

S. No.	Compound	Activity	Frequency	Score	Better	Equal	Worse	R group Distance	Docking Score	MMGbsa
1.	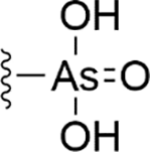	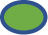	36	0.83	27	5	4	2.56	NIL	NIL
2.	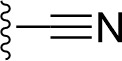	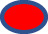	19	0.31	4	1	14	7.99	-6.927	-47.14
3.	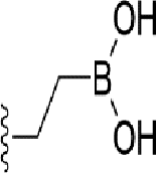	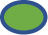	12	0.9	11	1	0	11.61	-6.928	-53.44
4.	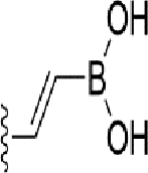	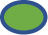	11	0.6	6	1	4	11.95	-6.952	-40.01
5.	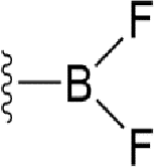	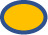	10	0.73	1	7	2	7.02	-6.308	-40.22
6.	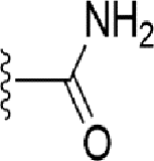	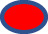	7	0.35	2	0	5	3.51	-6.654	-32.41
7.	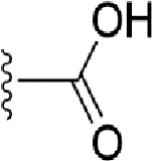	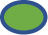	6	0.75	3	2	1	4.61	-4.946	-35.87
8.	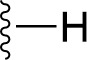	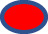	5	0.5	2	0	3	8.77	-4.258	-42.78
9.	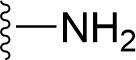	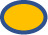	5	0.74	2	2	1	12.84	-5.913	-42.31
10.	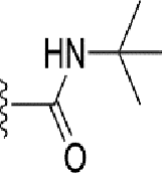	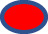	5	0.17	0	0	5	7.01	-4.201	-40.56
11.	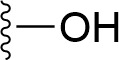	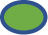	4	0.83	4	0	0	12.36	-4.894	-38.78
12.	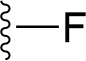	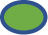	4	0.65	2	1	1	10.66	NIL	NIL
13.	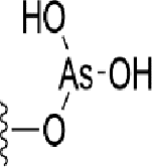	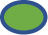	3	0.82	2	1	0	10.66	-4.159	-36.9
14.	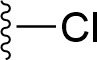	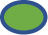	3	0.82	2	1	0	10.66	-4.786	-40.28
15.	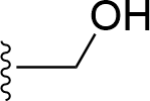	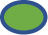	2	0.74	2	0	0	4.91	-5.253	-46.04
16.	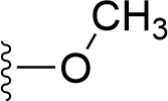	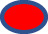	2	0.61	0	1	1	10.51	-6.07	-46.02
17.	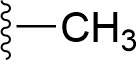	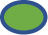	2	0.77	1	1	0	9.46	-4.775	-39.68
18.	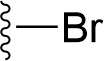	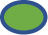	2	0.78	1	1	0	10.67	-5.117	-46.1
19.	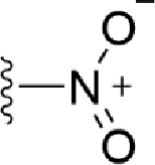	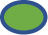	2	0.78	1	1	0	8.11	-4.52	-39.36
20.	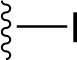	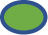	2	0.79	1	1	0	10.66	-6.183	-41.64
21.	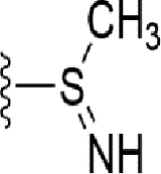	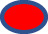	2	0.5	1	0	1	5.17	-5.868	-50.7
22.	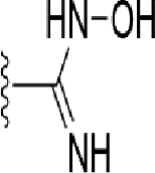	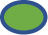	2	0.76	2	0	0	7.45	-5.844	-32.88
23.	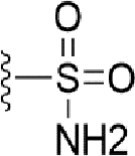	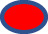	2	0.5	1	0	1	5.34	-6.205	-46.46
24.	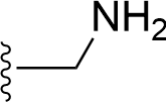	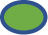	2	0.76	2	0	0	4.49	-4.861	-42.66
25.	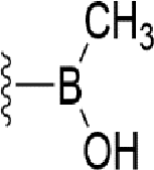	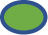	1	0.7	1	0	0	1.16	-5.915	-42.9
26.	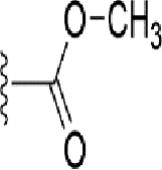	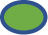	1	0.7	1	0	0	6.22	-5.807	-45.08
27.	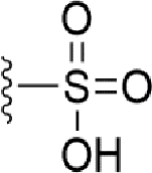	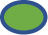	1	0.7	1	0	0	4.44	-5.627	-45.91
28.	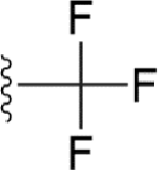	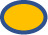	1	0.7	0	1	0	8.52	-6.949	-41.68
29.	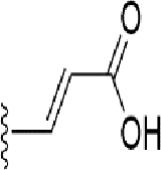	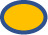	1	0.69	0	1	0	11.75	-6.074	-60.36
30.	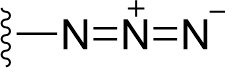	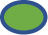	1	0.7	1	0	0	11.19	-5.849	-41.46
31.	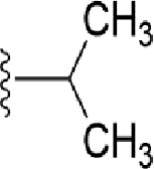	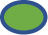	1	0.7	1	0	0	9.62	-6.634	-40.91
32.	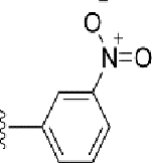	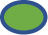	1	0.7	1	0	0	11.65	-6.923	-52.42
33.	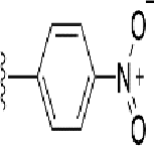	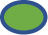	1	0.7	1	0	0	11.46	-6.105	-46.13
34.	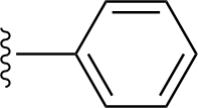	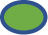	1	0.7	1	0	0	11.08	-6.728	-49.33
35.	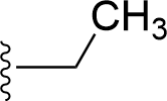	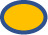	1	0.7	0	1	0	8.95	-4.417	-37.35
36.	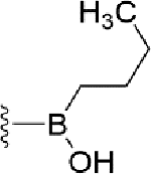	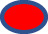	1	0.3	0	0	1	4.11	-6.784	-50.55
37.	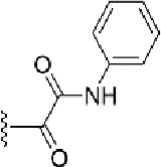	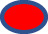	1	0.34	0	0	1	9.88	-6.447	-47.61
38.	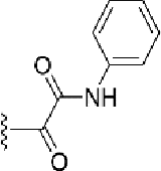	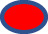	1	0.3	0	0	1	10.15	-6.685	-53.01
39.	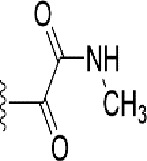	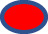	1	0.3	0	0	1	9.05	-6.075	-48.34
40.	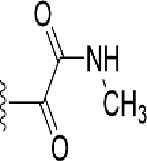	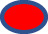	1	0.3	0	0	1	10.31	-7.217	-56.86
41.	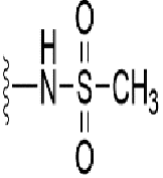	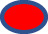	1	0.3	0	0	1	11.75	-6.669	-50.47
42.	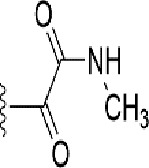	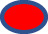	1	0.3	0	0	1	4.94	-5.843	-52.35

Docking score found between the range −4.0 and −6.7 with MMGBSA scores between −35.0 and −49.3 as estimated binding energy. MMGBSA defines the strength of binding between the ligand and the receptor; a greater value relates to the stronger binding. The activity of the replaced molecules is shown as nonpotentiating (RED color), may either potentiate or decrease the activity (ORANGE color), or potentiates the activity of the drug (GREEN color).

### Pharmacokinetic Assessment (ADMET)

**Figure 3:**
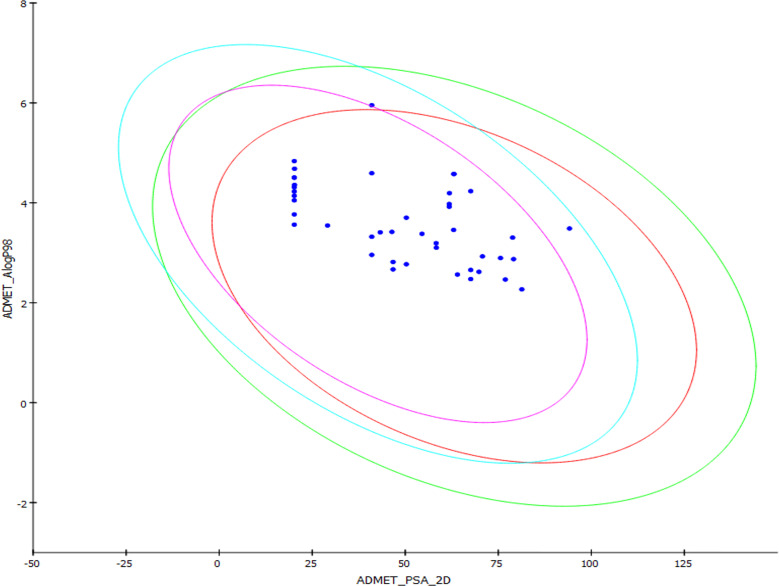
ADME/T evaluation plot of PSA *vs* AlogP for 38 lead molecules; 99% and 95% confidence limits correspond to intestinal absorption and blood–brain barrier models.

The drug-likeness of the newly generated bioisosteres was analyzed by Lipinski’s rule of 5. The majority of the compounds were below ALogP98, and 90% of the compounds have less hepatotoxicity. Twenty-four compounds displayed a good solubility, while the rest of the compounds had moderate solubility. In the case of adsorption and penetration, the majority of the generated compounds displayed potential for significant adsorption and penetration within a biological system. Overall, ADMET analysis revealed that 90% of the compounds generated in our study were more likely toward a potential drug (**Table 2**). Our interest molecule, 5-NPPP having an AlogP98 score of 3.418 and Pressure Swing Adsorption (PSA) of 46.42 was chosen. The presence of the functional (-NO_2_) group in 5-NPPP was evaluated further on chances of having a role in enhancing the activity when combined with antibiotics. When the ALogP98 was evaluated against the PSA, most of the isosteres lay within the effective adsorption area of the eclipse (**Figure 3**).

**Table 2:** ADMET prediction of the screened bio isosteric molecules.

S.No.:	Compound	AlogP98	Polar Surface Area (PSA)	Hepatotoxic	CYPD26	Aqueous Solubility	BBB Penetration	Absorption
1	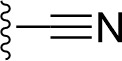	2.873	79.12	F	F	3	2	0
2	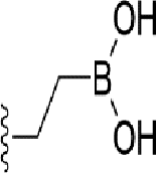	3.321	41.00	F	F	3	1	0
3	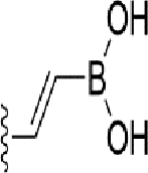	3.768	20.19	T	T	2	1	0
4	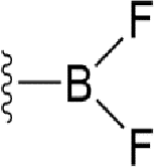	2.928	70.75	T	F	3	2	0
5	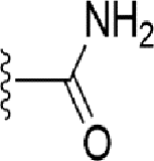	4.227	20.19	F	T	2	0	0
6	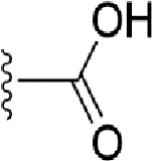	2.958	41.00	F	F	3	1	0
7	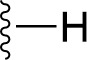	3.546	29.12	F	T	3	1	0
8	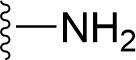	4.049	20.19	F	F	2	0	0
9	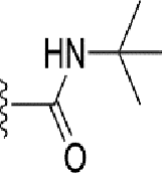	4.311	20.19	T	T	2	0	0
10	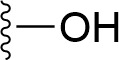	3.457	63.01	T	F	2	2	0
11	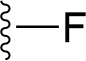	4.141	20.19	T	T	2	0	0
12	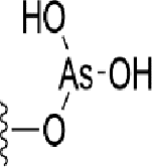	4.194	61.88	F	F	3	1	0
13	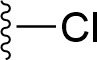	3.41	43.27	F	T	3	1	0
14	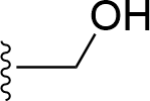	2.465	76.90	F	F	3	3	0
15	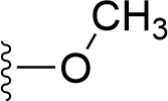	2.268	81.33	F	F	3	3	0
16	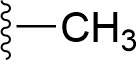	2.619	69.81	F	F	3	2	0
17	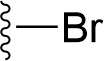	2.688	46.73	F	F	3	2	0
18	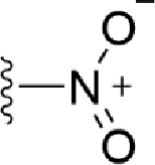	4.593	41.0	F	F	2	1	0
19	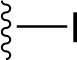	3.418	46.42	F	F	3	1	0
20	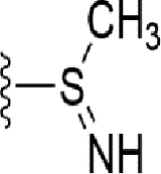	2.895	75.60	T	F	3	2	0
21	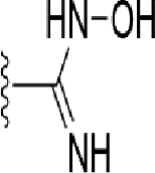	4.505	20.19	F	T	2	0	0
22	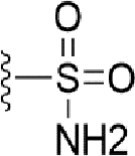	3.102	58.36	F	F	3	2	0
23	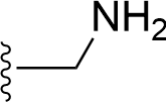	3.976	61.82	F	F	3	1	0
24	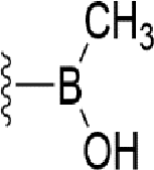	3.38	54.51	F	F	3	1	0
25	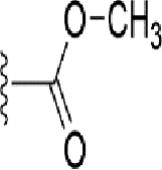	4.358	20.25	F	F	2	0	0
26	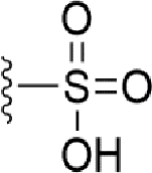	4.576	63.07	F	F	2	1	0
27	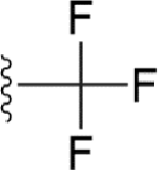	4.576	63.07	F	F	2	1	0
28	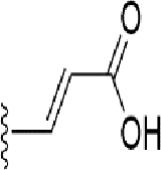	4.682	20.25	F	F	2	0	0
29	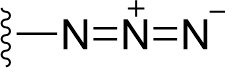	4.505	20.19	F	T	2	0	0
30	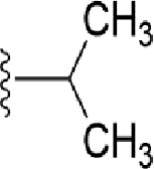	5.953	41.0	F	F	2	0	0
31	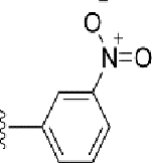	4.233	67.60	T	F	2	1	0
32	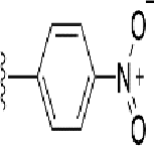	3.304	78.86	F	F	3	2	0
33	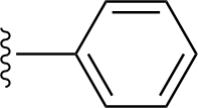	2.656	67.60	T	F	3	2	0
34	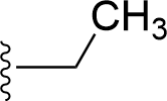	4.835	20.19	F	F	2	0	0
35	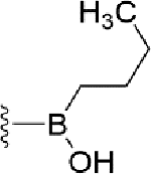	3.846	94.19	T	F	2	3	0
36	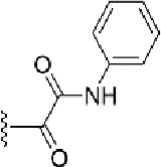	2.474	67.60	T	F	3	2	0
37	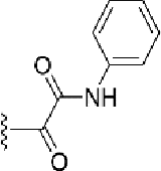	2.771	50.30	T	F	3	2	0
38	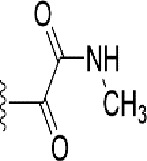	2.565	64.03	T	F	3	2	0
39	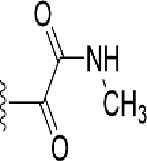	3.192	58.30	T	F	3	2	0
40	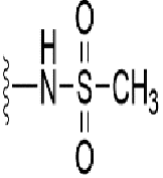	3.563	20.19	F	T	3	1	0
41	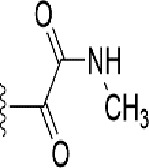	2.816	46.73	T	F	3	2	0
42	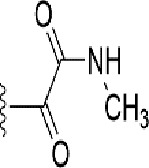	3.702	50.30	T	F	2	1	0

AlogP98 (≤ 2.0 or ≥ 7.0)—very poor absorption; PSA (≥ 150)—very low absorption; hepatotoxicity (T—True, F—False); CYPD26 (T—inhibitor, F—non-inhibitor); aqueous solubility (0—low; 1—very low, but possible; 2—low solubility; 3—good solubility; 4—optimal soluble; 5—too soluble); BBB penetration (0—very high penetration; 1—high penetration; 2—medium penetration; 3—low penetration; 4—undefined); absorption (0—good; 1—high; 2—medium; 3—low; 4—undefined).

### Identification of *S. aureus* Clinical Strain Overexpressing NorA

Clinical strains of *S. aureus* were screened for resistance/sensitivity toward the broad spectrum of antibacterial agents that belongs to quinolones, fluoroquinolones, macrolide, penicillin, and cephalosporins. Among the clinical isolates, *Sa*-P1920, *Sa*-P2003, and *Sa*-EYE33 exhibited a multidrug resistance (MDR) phenotype. Also, a key observation was that the strain *Sa*-P2003 exhibited resistance to fluoroquinolones such as CIP and NOR (**Figure S1**). An earlier report suggests the involvement of the NorA efflux pump expelling fluoroquinolones and favoring the pathogens to acquire intrinsic resistance. So, we intend to explore the interrelationship of the NorA efflux pump expression by the MDR clinical isolates. The data were quite significant to show that the clinical isolate *Sa*-P2003 was observed to overexpress *norA*, as the cycle threshold (CT) value was several folds lesser in comparison with the other strains (**Figure S2**). The data were also in concordance with the EtBr efflux activity (data not shown).

### Combinatorial Action of 5-NPPP With CIP

**Figure 4:**
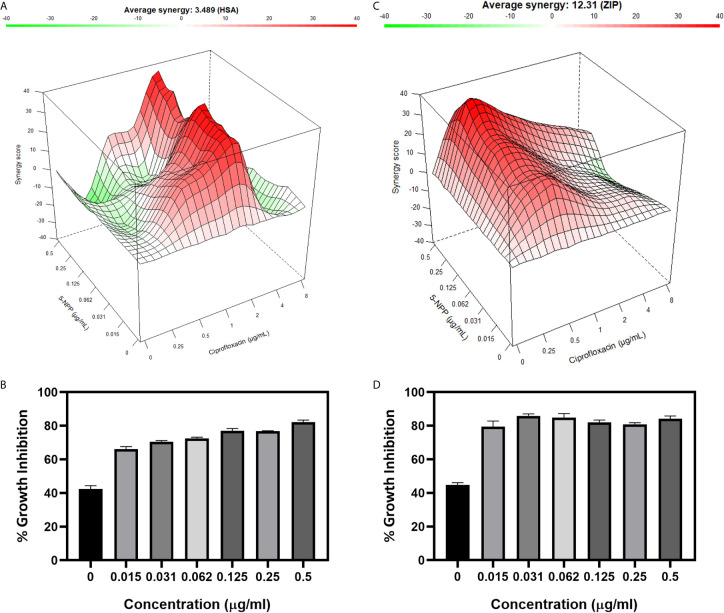
Effect of 5-NPPP in enhancing the bactericidal potential of ciprofloxacin. **(A)** Synergy plot of *Sa*-P2003 at varying concentrations of ciprofloxacin and 5-NPPP. The synergy score of 3.489 (Highest Single Agent model) shows additive. **(B)** For *Sa*-P2003, at the constant concentration of ciprofloxacin (0.5 µg mL^−1^), the concentration-dependent growth inhibitory activity of 5-NPPP is shown. **(C)** Synergy plot of SA-1199B at varying concentrations of ciprofloxacin and 5-NPPP. The zero-interaction potency model was used to fit the synergy data having the synergy score of 12.31, which shows synergism. **(D)** For SA-1199B, at the constant concentration of ciprofloxacin (0.5 μg mL^−1^), the potentiation activity of 5-NPPP was established at the minimum concentration of 0.015 µg mL^−1^ (5-NPPP).

The potentiation activity of 5-NPPP with CIP was assessed using a checkerboard assay. The MIC of CIP against the strains SA-1199B and *Sa*-P2003 was evaluated to be 8 µg/ml. In the combination with 5-NPPP, the concentration of CIP against these two strains (**Figures 4B, D**) was drastically reduced to 0.5 µg/ml, having 16-fold reduction in MIC, thus proving its potentiating efficacy. In addition, the combinatorial action of 5-NPPP and CIP was analyzed further by fitting into the ZIP model (**Figure 4A**). The ZIP model overcomes the drawbacks of other drug interaction models wherein the combined response does not affect the individual potency of the drugs. From the response plot, it can be observed that CIP showed a strong additive effect (score of 3.49) with 5-NPPP for *Sa*-P2003 (**Figure 4A**), whereas a strong synergism score of 12.31 was achieved for SA-1199B (**Figure 4C**). The scores are based on the cumulative evaluation of all the concentrations considered. A closer look reveals a 16-fold reduction in the ciprofloxacin concentration against both the strains. Thus, 5-NPPP significantly increases the bactericidal activity of CIP than the effect of either drug against *Sa*-P2003.

### Evaluation of the 5-NPPP on EtBr Efflux Action

**Figure 5:**
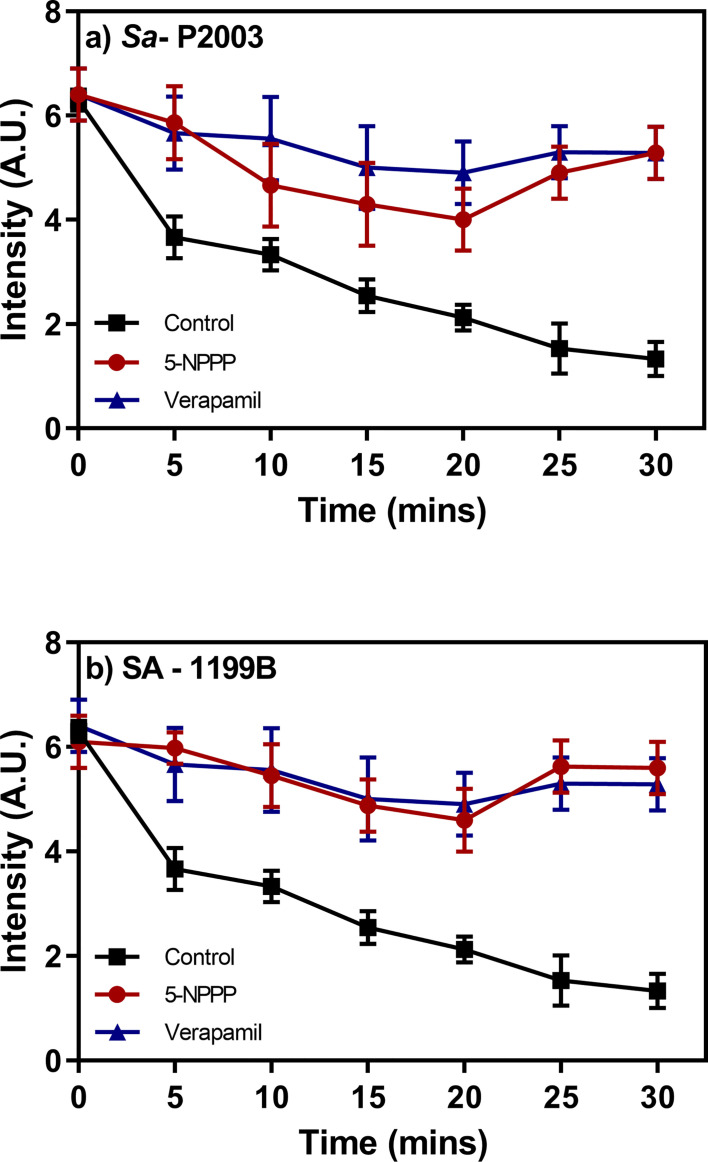
Evaluation of the effect of the identified NorA efflux pump efflux and the accumulation of ethidium bromide in **(A)** *Sa*-P2003 and **(B)** SA-1199B. Verapamil is included as positive control, and the efflux activity without the addition of any drug is considered as a negative control.

The clinical isolates *Sa*-P2003 and SA-1199B as independent trials were loaded with the known substrate (EtBr) of the NorA efflux pump either with or without 5-NPPP. The samples were placed in a fluorometer cuvette containing a fresh nutrient medium, and the assay was carried out for 30 min. There was a time-dependent decrease in the fluorescence that was observed due to the action NorA-mediated efflux of EtBr as maximal extrusion was observed in the control (untreated). On contrary, the cells treated with 5-NPPP showed prolonged retention of fluorescence, and the data were equivalent to the known EPI verapamil. In both the strains, ~76% reduction in the EtBr efflux was achieved by 5-NPPP treatment (**Figure 5**). A similar and comparable EtBr efflux reduction was achieved for the positive control, verapamil. The observed data suggest that, in the absence of 5-NPPP, the EtBr readily effluxes out of the cells *Sa*-P2003 and SA-1199B due to the action of the NorA as it is not favored to intercalate onto the DNA.

### Evaluation of Cytotoxicity

**Figure 6:**
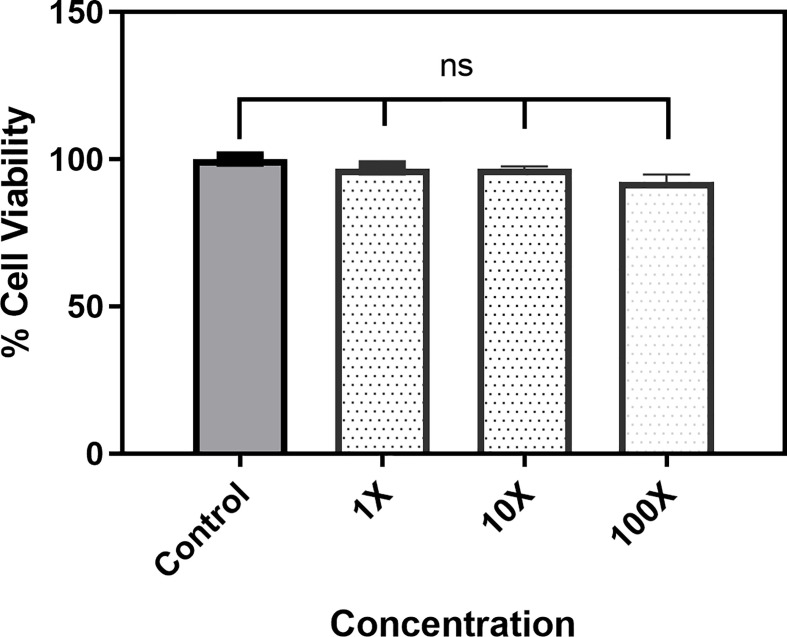
Cytotoxicity assay. The bar graph depicts the viability of the Hep-G2 in the presence of 1X, 10X, and 100X of the 5-NPPP. ns, non-significant.

The effect of 5-NPPP on host mammalian cells was analyzed using Hep-G2 cells. Three different concentrations, 1X MIC (400 μg mL^-1^), 10X (4 μg mL^-1^), and 100X (40 μg mL^-1^), were tested, and the data revealed that, even at high concentration, 5-NPPP was nontoxic to Hep-G2 (**Figure 6**). Hence, 5-NPPP provides a possibility for the development of the nontoxic active molecule that could increase the antibacterial effect of fluoroquinolones against *S. aureus.*

## Discussion

The efficacy of boronic acid and its derivatives as effector molecules for the development of therapeutic agents has been well established (Albers et al., 2011; Maynard et al., 2014; Hecker et al., 2020). As a part of therapeutic potential, the antibacterial effect of boronic acid was known to suppress the production of virulence determinants and antibiotic-degrading enzymes to enhance the activity of antibiotics (Trippier and McGuigan, 2010). Similarly, a wide range of boronic acid derivatives has been explored for their therapeutic potential as *β*-lactamase inhibitors against class A and class C *β*-lactamases (Crompton et al., 1988; Tondi et al., 2010; Winkler et al., 2013; Cendron et al., 2019). Boronic acid transition state analogue inhibitors have been effective in subduing the *β*-lactamase-based resistance in *P. aeruginosa* (Eidam et al., 2012). Vabomere^®^, a drug composed of boronic acid and the *β*-lactam meropenem, has been marketed to treat urinary tract infections caused by (MDR) *Pseudomonas* and carbapenem-resistant *Enterobacteriaceae* (Griffith et al., 2019). Pentapeptide boronic acid inhibitors were effective against the MycP_1_ protease of the Esx secretion system, one of the major virulence determinants of *Mycobacterium tuberculosis* (Frasinyuk et al., 2014; Cao et al., 2015; Bosserman and Champion, 2017). Recently, several pyridine-3-boronic acids were screened against NorA overproducing mutant *S. aureus* strain SA-1199B, and the pyridine-3- and benzene boronic acids were reported to be the most effective against NorA. The data also suggest that 6-benzyloxypyridine-3-boronic acid and 4-benzyloxybenzene boronic acid enhanced the antibacterial activity of CIP with minimum modulatory concentration (MMC) of 1.0 and 0.5 μg/ml, respectively (Fontaine et al., 2014). Further chemical pharmacomodulation revealed that 6-benzyloxypyridine-3-boronic acid could increase CIP activity by fourfold at 16 μg/ml against the NorA overexpressing strain, SA-1199B (Fontaine et al., 2015). However, some groups of boronic acids including the heterocyclic, cyclopropyl, and vinyl boronic acids have been reported to be unstable undergoing decomposition upon exposure to air through the process of oxidation, polymerization, and protodeboronation (Knapp et al., 2009). Based on these evidences, 6-benzyloxypyridine-3-boronic acid was chosen as the pharmaco-template for our study. In the present study, we intended to replace the boronic acid functional group from the template molecule using the bioisosteric approach to enhance the CIP potentiating activity from fourfold to several-fold. In this context, we could build about 42 replacement molecules through the Swiss Bioisostere tool. The 42 compounds that were generated were analyzed for the potential to interact with the NorA efflux pump by docking studies and their property for drug-likeness. The results indicate that many isosteres of the parent compound do interact with the target, and the ligands do possess significant solubility and adsorption potential. Further evaluation of their potential to get absorbed into the biological system revealed that about 39 compounds could be well absorbed into the human gut, and two molecules were known to cross the blood–brain barrier. For our current study, we have taken forwarded 5-NPPP having the nitro group in the place of the boron group by considering two aspects—ease of synthesis and biological significance. In the synthesis perspective, nitro groups can be introduced easily *via* any one of the following methods: (i) C-H nitration (Song et al., 2019), (ii) nitration in ionic liquid (Moosavi-Zare et al., 2016), (iii) copper-catalyzed mild nitration (Hernando et al., 2014), (iv) using a mild tert‐butyl nitrite as the nitro source (Li et al., 2019), and (v) oxidation of aromatic amines (Ravi et al., 2019). Additionally, as these nitration processes are tolerant with various functional groups, the nitro group can be introduced to any biologically interesting core molecules without any change in the core structure (Kilpatrick et al., 2013).

The *in silico* ADMET studies revealed that 5-NPPP exhibited an AlogP98 score of 3.418 and PSA of 46.42 was chosen. Earlier studies predicted that the designing of NorA specific drugs targeting the amino acids Trp or Met or Phe has been suggested to modulate protein function effectively (Ma and Nussinov, 2007). Similarly, in our study, 5-NPPP interacts with the NorA protein in the phenylalanine group. Moreover, the interaction also occurs in the π–π interaction pattern, suggesting the exertion of van der Waal’s forces to act upon the complex formed between the ligand and the target (**Figure 2**). A previous study has demonstrated that having various kinds of intermolecular interactions such as van der Waal’s, alkyl-, π-sulfur, π–π, and π-alkyl is found to be responsible for the stability of the NorA complex with the ligands (Figueredo et al., 2020). The nitro group allows electron donation property, allowing further modulation of intracellular oxidative stress (Benites et al., 2010). Similarly, from the class of the indole group, the compound 5-nitro-2 phenylindole (INF55) was able to potentiate the action of CIP (1.5 µg/mL) with a fourfold increase against *S. aureus* (Markham et al., 1999). Modifying the structures at positions of C2, C3, and C5 in the primary discovered compounds has always preserved an electron-withdrawing group such as nitro in 1, 3, 4 or cyano group in 2 (Ambrus et al., 2008; Samosorn et al., 2009). It is interesting to note that indole-carrying halogen atoms in the C5 position and nitrone moiety in C3 are found to possess better EPI activity (Hequet et al., 2014). Nitro groups are known to resemble pro-drug, and reduction of nitro groups by enzymatic process leads to the formation of reactive species and exhibits therapeutic effects against bacterial pathogens (Wardman, 2001). A study has shown the reduction in the therapeutic potential of flutamide in the treatment of prostate cancer when the inherent nitro moiety of the drug was replaced with cyano (Coe et al., 2007). Similarly, when the nitro moiety was replaced with acetyl, benzoyl, methyl sulfone, or amide in the FDA-approved anthelmintic agent, niclosamide, the biological activity was significantly modified (Mook et al., 2015). Hence, the presence of the nitro group in the identified lead molecule, 5-NPPP, could potentially serve in resensitizing multidrug resistant *S. aureus* strains that overexpress the efflux pump NorA.

In this “post-antibiotic” era, several pathogens have developed multidrug resistance to most of the conventional antibacterial and antifungals, which leads to the failure of treatment. Bacterial cells express antibiotic resistance through various mechanisms, and one such mechanism is through effusing the antibacterial agents across the outer membrane by efflux pumps. About 10 plasmids or chromosome encoded multidrug efflux pumps have been defined in *S. aureus*, some of which can expel structurally diverse antibacterial agents (Piddock, 2006; Costa et al., 2013). Thus, the development of EPI, which blocks the efflux pump action, is essential to overcome the antibiotic resistance mechanisms. The major efflux pumps of *S. aureus* including NorA, NorB, NorC, SdrM, MdeA, LmrS, QacA, QacB, Tet38, and TetA (K) belong to the MFS (Yoshida et al., 1990; Huang et al., 2004; Truong-Bolduc et al., 2005; Truong-Bolduc et al., 2006; Yamada et al., 2006; Lekshmi et al., 2018). Among these multidrug efflux pumps, *norA* confers *S. aureus* the ability to resist the antibacterial effect of hydrophilic quinolones such as CIP, NOR, ofloxacin, and enoxacin, and less or complete susceptibility to hydrophobic quinolones (Yoshida, 1992). When a strain overexpresses *nor*A, a high level of NorA protein accumulates in the cytoplasmic membrane. Thereby, *S. aureus* strains resist fluoroquinolones (Kaatz and Seo, 1995). In this regard, the effluxing activity of the overexpressing strains was significantly reduced in the presence of 5-NPPP (76% inhibition), which is comparable to the positive control, Verapamil (Aeschlimann et al., 1999). This proves that our modification has enhanced the efflux pump inhibitory effect. Moreover, fluoroquinolones including CIP and NOR are suitable substrates for NorA protein (Singh et al., 2017). Alteration in the expression of *norA* connects intrinsic CIP resistance and evolvability. Suppressing NorA in the clinical strains had resulted in the borderline loss of resistance in *S. aureus* 16(Papkou et al., 2020). Many chemically diverse NorA inhibitors that synergistically increase the bactericidal efficacy of CIP in NorA overexpressing *S. aureus* strain had been reported. In this regard, 5-NPPP was shown to reduce the concentration of CIP 16-fold. The MIC of 0.5 µg/ml was achieved by the presence of 5-NPPP, thus proving the enhanced potentiating activity of 5-NPPP. Furthermore, our study has demonstrated that, at very minimal concentration without toxicity to mammalian cells, 5-NPPP in combination with CIP increased the susceptibility of MDR *Sa*-P2003 and SA-1199B through inhibition of the efflux pump NorA.

Thus, the current work identifies a novel derivative of boronic species 5-NPPP as the effective EPI against the NorA overexpressing strains. The preliminary studies on 5-NPPP effectively prove the significant reduction in the EtBr efflux, the potentiating activity of the CIP to 16-fold reduction, and minimal mammalian cell toxicity. This paves the path to explore this compound further in terms of the pharmacokinetic and pharmacodynamic studies. Future studies are under steady progress to exploit 5-NPPP in *in vivo* infectious models.

## Data Availability Statement

The original contributions presented in the study are included in the article/**Supplementary Material**. Further inquiries can be directed to the corresponding author.

## Author Contributions

Study conception design and project administration: APS. Computational data analysis and interpretation of results: GT, AS, and KS. 5-NPPP synthesis and characterization: GT and PV. In vitro data acquiring: GT. In vitro data analysis and interpretation of results: GT and APS. Original draft manuscript preparation, writing, reviewing, and editing: GT, HB, SV, and APS. All authors have read and agreed to publish this version of the manuscript.

## Conflict of Interest

The authors declare that the research was conducted in the absence of any commercial or financial relationships that could be construed as a potential conflict of interest.

## Publisher’s Note

All claims expressed in this article are solely those of the authors and do not necessarily represent those of their affiliated organizations, or those of the publisher, the editors and the reviewers. Any product that may be evaluated in this article, or claim that may be made by its manufacturer, is not guaranteed or endorsed by the publisher.
