# Supplementary material for: Development of an Antibiotic Resistance Breaker to Resensitize Drug-Resistant Staphylococcus aureus: In Silico and In Vitro Approach
Source: Front Cell Infect Microbiol. 2021 Aug 16;11:700198. doi: 10.3389/fcimb.2021.700198 (PMC8415528; doi:10.3389/fcimb.2021.700198)
Supplement: Supplementary file 1 [file DataSheet_1.docx]

**Supplementary File**

**Development of an antibiotic resistance breaker to re-sensitize drug-resistant *Staphylococcus aureus*: *in silico* and *in vitro* approach.**

Gopalakrishnan T^1^,Hema Bhagavathy Sarveswari ^1^, Sahana Vasudevan^1^, Karthi Shanmughan^1^, Alex Stanley ^1^, Pothiappan Vairaprakash^2^, and Adline Princy Solomon*^1^.

Figure S1. Resistant pattern (%) of *S. aureus* isolates from infected outpatients’ year 2015-2016 (JSS Medical University, Mysore). VAN (Vancomycin), CEF (Cefoxitin), NOR (Norfloxacin), CEP (Cephalexin), CIP (Ciprofloxacin), CHL (Chloramphenicol), AZI (Azithromycin), CLO (Cloxacillin)


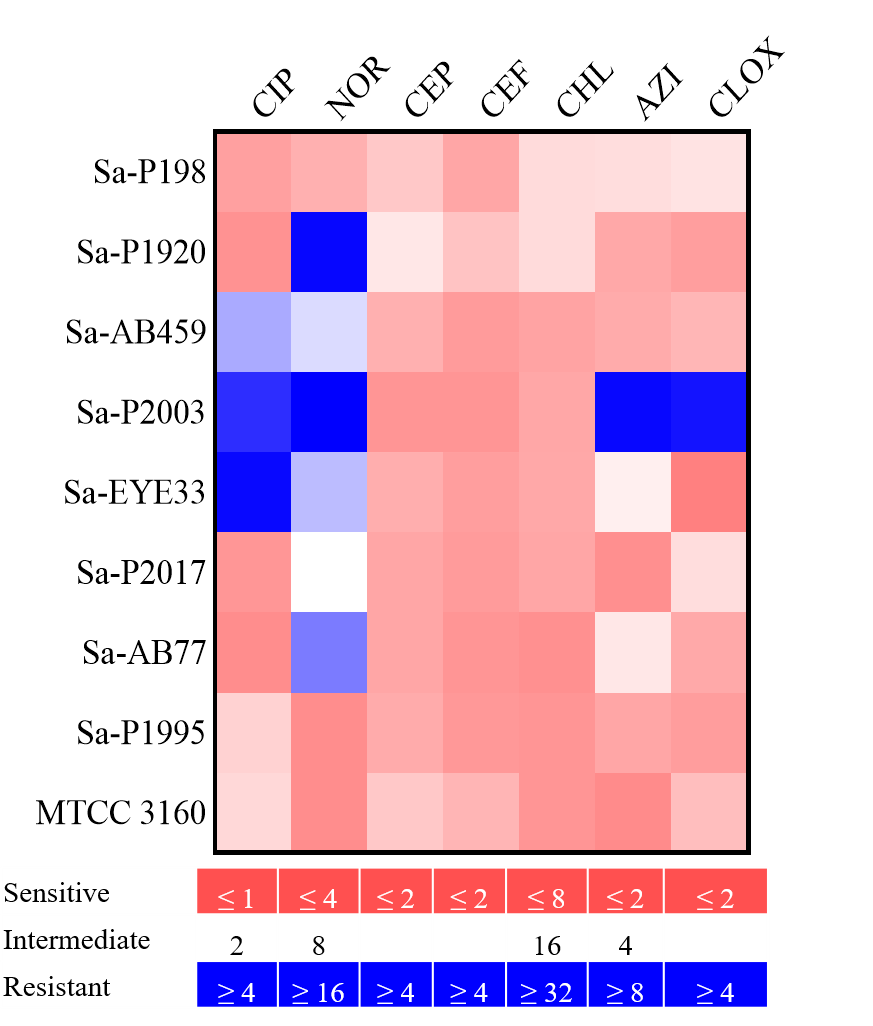


**Figure S2. Overexpression of *norA* by *Staphylococcus aureus* strains isolated from various clinical specimens.**


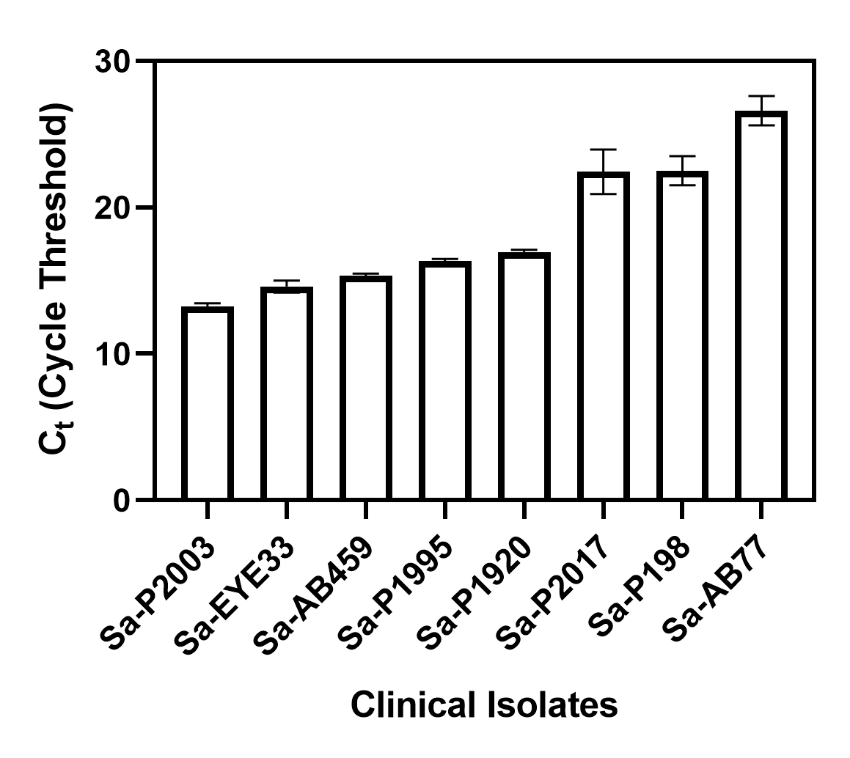


**Table S1: Primers used in the study**

| Gene Name | Gene Function | Forward Primer Sequence | Reverse Primer Sequence | Reference |
| --- | --- | --- | --- | --- |
| *norA* | membrane-based multidrug efflux transporter | TTTGTTTTCAGTGTCAGAATTTATGTTTG | GGCTTGGTGAAATATCAGCTATTAAAC | Iman *et al.*  2020 |
| *gmk-rt* | Guanylate cyclase- | TATCAGGACCATCTGGAGTAGG | CATCAACTTCACCTTCACGC | Pourmand *et al.* 2014 |
